# A Potential Novel Mechanism for Vagus Nerve Stimulator-Related Central Sleep Apnea

**DOI:** 10.3390/children4100086

**Published:** 2017-09-29

**Authors:** Inga C. Forde, Meghna P. Mansukhani, Bhanu Prakash Kolla, Suresh Kotagal

**Affiliations:** 1 Center for Sleep Medicine, Mayo Clinic, 200 First Street SW, Rochester, MN 55905, USA; forde.inga@mayo.edu (I.C.F.); mansukhani.meghna@mayo.edu (M.P.M.); 2 Center for Sleep Medicine and Department of Psychiatry and Psychology, Mayo Clinic, 200 First Street SW, Rochester, MN 55905, USA; kolla.bhanuprakash@mayo.edu; 3 Center for Sleep Medicine and Division of Pediatric Neurology, Mayo Clinic, 200 First Street SW, Rochester, MN 55905, USA

**Keywords:** vagus nerve stimulator, epilepsy, sleep disordered breathing, mechanisms

## Abstract

The treatment of epilepsy with vagus nerve stimulation can inadvertently cause obstructive and central sleep apnea (CSA). The mechanism for CSA seen in patients with a vagus nerve stimulator (VNS) is not fully known. We describe the case of a 13-year-old girl in whom VNS activation induced tachypnea and post-hyperventilation central apnea. Following adjustment of VNS settings, the post-hyperventilation CSA resolved. Polysomnography may assist with management when patients with epilepsy develop sleep disruption after VNS placement.

## 1. Introduction

Vagus nerve stimulators, implanted for the treatment of refractory epilepsy, have been associated with obstructive, and less commonly, central sleep apnea (CSA). The pathophysiology of vagus nerve stimulator (VNS)-mediated CSA is not known. In this report, we describe a possible pathophysiologic mechanism for CSA secondary to a VNS.

## 2. Case Presentation

A 13-year-old girl was evaluated for suspected sleep-disordered breathing (SDB). She was born at term, following a normal pregnancy, and achieved adequate early milestones. At 33 months of age, she developed febrile status epilepticus; the seizures were stabilized on valproic acid, which was then weaned off by age 36 months. The seizures recurred at the age of 6 years and remained refractory to multiple antiepileptic drugs. At the age of 12 years, a VNS was implanted for enhancing seizure control.

A year later, the patient’s mother noticed an altered breathing pattern—the patient would take intermittent deep breaths in her sleep followed by pauses lasting up to 10 seconds. Breathing was described as “noisy”. The patient herself did not have any sleep complaints. Her medications included Adderall XR™ 25 mg daily for attention deficit hyperactivity disorder, melatonin 0.5 mg at bedtime for insomnia, ethosuximide 250 mg daily and lacosamide 75 mg twice daily for seizure control, with midazolam 7.5 mg intranasally as a “rescue” medicine for seizures. The patient’s body mass index was 18.4 kg/m^2^, just below the 40th percentile for age. There was no craniofacial anomaly or tonsillar hypertrophy. A nocturnal polysomnogram (PSG) was ordered to assess sleep-related breathing function; VNS settings at that time are shown in Table 1.

**Figure 1:**
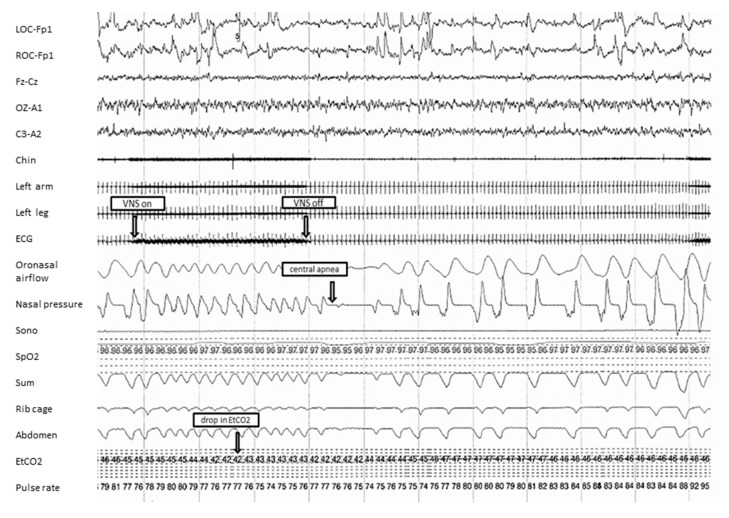
A representative 120-s fragment from the first overnight diagnostic polysomnogram. The figure shows tachypnea at the time of vagus nerve stimulator (VNS) activation, followed by central apnea. VNS-on and VNS-off were identified based on artifact observed in the electrocardiogram (ECG) lead. Mean end-tidal carbon dioxide ranged between 20–52 mm Hg, with 0.2% of the total time spent with an end-tidal carbon dioxide level greater than 50 mm Hg. Sono: sonogram; SpO2: oxyhemoglobin saturation (in %); Sum: summation channel of thoracic and abdomen inductance plethysmography; EtCO_2_: end-tidal carbon dioxide (in mm Hg).

As illustrated in Figure 1, intermittent activation of the VNS during sleep was seen to trigger episodes of tachypnea that led to drops in the end-tidal carbon dioxide from a baseline of 47 mm Hg to 42 mm Hg, with consequent post-hyperventilation central apnea.

Sleep parameters during the first polysomnogram at our center are shown in Table 2. During all of the periods of VNS activation, hyperventilation was noted, and each of these hyperventilation episodes was seen to result in a central apnea lasting 10 s or longer. Mean duration of central apneas was 15 s. There were no significant central sleep disordered breathing events that occurred independent of VNS stimulation. Central apneas were seen in all stages and positions of sleep. Approximately 75% of central apneas were accompanied by arousals which could potentially increase the predilection for seizures [1]. The vast majority of central sleep disordered breathing events were not associated with significant desaturation. Our patient was diagnosed with mild CSA.

The stimulus intensity was decreased to 1.25 milliamps on a repeat PSG three months later (revised VNS settings are shown in Table 1), with a corresponding decrease in the VNS activation-associated tachypnea, noted as a resolution of the hyperventilation episodes after VNS stimulation. The mother did not note any abnormal breathing patterns at night and seizure control did not change significantly after the VNS setting changes. On the second polysomnogram (sleep parameters are shown in Table 2), some obstructive sleep disordered breathing events were seen in conjunction with VNS activation. Short central apneas were also noted. These were independent of VNS activation and not associated with significant desaturation or arousals.

## 3. Discussion

Vagus nerve stimulation was approved by the Food and Drug Administration for the treatment of epilepsy in 1997 [2]. The mechanism by which the VNS device improves seizure control is not completely known; it may involve an alteration in synaptic circuitry and inhibition of epileptogenic potentials [3]. Reported adverse effects of vagus nerve stimulation to date include dysphonia, dyspnea, laryngeal irritation, stridor, and unilateral vocal cord palsy, as well as obstructive sleep apnea (OSA) and CSA [2,4,5,6].

The pathophysiology of CSA in epilepsy patients with VNS implants has not been fully elucidated [7]. An increased respiratory frequency associated with decreased amplitude of breaths has been described in patients with a VNS [8]. Activation of the ventral respiratory group of neurons located close to the nucleus ambiguus-retroambigualis in the ponto-medullary region is known to alter respiratory rhythm [9]. It is possible that such a disturbance may have occurred due to VNS activation in our patient, leading to post-hyperventilation apnea. VNS-induced tachypnea may override the patient’s baseline respiratory pattern and potentially result in diaphragmatic fatigue. Once the VNS stimulation ceases, it may take some time for diaphragmatic function to recover and for breathing to resume, resulting in central apnea. In this case, central apnea occurred after, and not during VNS activation, which, to our knowledge, has not been reported previously.

Manipulation of VNS settings such as stimulus intensity, frequency, and cycle times may decrease the respiratory disturbance [10]. However, the specific stimulus parameter adjustment that would be most effective in ameliorating the VNS-related respiratory arrhythmia is not known at this time. Short respiratory pauses of less than 10 s seen in this setting that are unaccompanied by significant bradycardia and/or gas exchange abnormalities may be benign and not of much clinical significance. Such events, if noted on polysomnography following VNS stimulation, may not necessitate a change in VNS settings.

## 4. Conclusions

This case demonstrates that triggering of a VNS device may result in tachypnea and subsequent post-hyperventilation central apnea. PSG should be considered after the implantation of a VNS if there are increased nighttime arousals in order to assess for SDB. Adjustment of VNS settings may be required if the onset of the respiratory disturbances coincides with the activation of the VNS.

## Figures and Tables

**Table 1:** Vagus nerve stimulator settings.

Settings	Before the First PSG	Before the Repeat PSG
Output Current (mA)	1.5	1.5
Signal frequency (Hz)	30	30
Pulse width (microseconds)	500	500
Signal ON time (seconds)	30	30
Signal OFF time (minutes)	1.8	1.1
Magnet output current (mA)	1.75	1.25
Magnet ON time (seconds)	60	60
Magnet pulse width (microseconds)	500	250

PSG: polysomnogram.

**Table 2:** Sleep parameters during the first and second polysomnograms.

Sleep Parameter	First PSG	Repeat PSG
Total sleep time (minutes)	447.2	301
Sleep efficiency (%)	90.3	62.3
Apnea-hypopnea index (per hour)	2	6
Respiratory disturbance index (per hour)	3	6
Central apnea index (per hour)	2	3
NREM supine sleep time (minutes)	75.4	179.5
AHI in NREM supine sleep (per hour)	2	6
NREM nonsupine sleep time (minutes)	328.3	100.5
AHI in NREM nonsupine sleep (per hour)	2	6
REM supine sleep time (minutes)	14	0
AHI in REM supine sleep (per hour)	0	-
REM nonsupine sleep time (minutes)	29.5	21
AHI in REM nonsupine sleep (per hour)	4	3
Mean oxyhemoglobin saturation (%)	96	96
Minimum oxyhemoglobin saturation (%)	93	92
Arousal index (per hour)	10.6	7
Breathing-related arousals (%)	25.3	22.9
Periodic limb movement index (per hour)	0	1

AHI: Apnea-hypopnea index; NREM: non-rapid eye movement sleep; REM: rapid eye movement sleep.
